# It’s about TIME – Gal-9 as a potential immunotherapeutic target in pancreatic ductal adenocarcinoma

**DOI:** 10.3389/fimmu.2025.1495907

**Published:** 2025-01-31

**Authors:** Christin Knickmeier, Gaetan Aime Noubissi Nzeteu, Bernhard F. Gibbs, Frederik J. H. Hoogwater, Maarten W. Nijkamp, Maximilian Bockhorn, N. Helge Meyer

**Affiliations:** ^1^ Department of Human Medicine, School of Medicine and Health Sciences, Carl von Ossietzky Universität Oldenburg and University Hospital for General and Visceral Surgery, Oldenburg, Germany; ^2^ School of Psychology and Life Sciences, Canterbury Christ Church University, Canterbury, United Kingdom; ^3^ Section of HPB Surgery and Liver Transplantation, Department of Surgery, University Medical Center Groningen, University of Groningen, Groningen, Netherlands

**Keywords:** pancreatic ductal adenocarcinoma, tumor immune microenvironment, immunotherapy, immune checkpoints, galectin-9, myeloid-derived suppressor cells, macrophage (re-)polarization

## Abstract

Pancreatic ductal adenocarcinoma (PDAC) remains one of the most lethal malignancies, characterized by an extremely poor prognosis and limited therapeutic options. Central to the progression and immune evasion of PDAC is the tumor (immune) microenvironment (TIME), where immune checkpoint proteins such as galectin-9 (Gal-9) play pivotal roles. Gal-9 significantly contributes to the immunosuppressive milieu of PDAC by interacting with various immune cells, such as T cells, macrophages, and myeloid-derived suppressor cells (MDSCs). These interactions suppress anti-tumor immunity, thus facilitating tumor growth and metastasis. This review comprehensively examines the multifaceted role of Gal-9 in the TIME of PDAC, detailing its mechanisms of action, including the induction of regulatory T cells, polarization of tumor-associated macrophages, and modulation of apoptotic pathways *via* Tim-3 and caspase activation. The therapeutic potential of targeting Gal-9, either alone or in combination with other immune checkpoint inhibitors such as anti-PD-L1, is also discussed, highlighting preclinical findings that suggest promising avenues for enhancing anti-tumor immune responses. By elucidating the complex biological activities of Gal-9 and its interactions within the TIME, this review underscores the importance of innovative therapeutic strategies aimed at mitigating the immunosuppressive effects of Gal-9 in PDAC.

## 1 Background

### 1.1 Pancreatic ductal adenocarcinoma

Pancreatic ductal adenocarcinoma (PDAC) is a significant global health burden, being the third leading cause of cancer-related deaths in the United States with continued increase in incidence and mortality. On a global scale, data from 2022 also reflect the pervasive impact of PDAC – being the 6^th^ leading cause of cancer death, emphasizing the need for enhanced diagnostic and therapeutic strategies. According to recent statistics on PDAC, the five-year overall survival rate for PDAC in the USA is currently only 13%, reflecting the aggressive nature and late-stage diagnosis common with this cancer. For 2024, it is projected that there will be approximately 66,440 new cases of PDAC in the USA, with an estimated 51,750 deaths attributed to the disease. These figures underscore the critical need for advancements in early detection and treatment strategies (1). The prognosis for PDAC is particularly poor, especially in cases where the disease has metastasized. At the time of initial diagnosis, approximately 50% of PDAC patients present with synchronous distant metastases. A recent study analyzed data from the SEER database and confirmed that, at the initial diagnosis, many PDAC patients present with metastases, primarily in the liver which significantly impacts their prognosis. These patients have a drastically reduced overall survival rate, often less than 6 months, compared to those without metastases, whose median survival can extend beyond a year.

### 1.2 Galectin-9

Galectin-9 (Gal-9) is a 36 kDa protein with its two carbohydrate recognition domains (CRDs), connected by an unstructured linker peptide (2, 3). These CRDs form glycan-binding pockets, which confer different affinities and biological activities depending on their glycan partners (3, 4). The structural specificity of these CRDs is crucial, as variations significantly influence receptor recognition, signaling pathways, and cell death mechanisms in immune responses (4, 5). In normal physiology, Gal-9 is involved in various cellular functions, including cell aggregation, adhesion, and apoptosis (6). Gal-9 is expressed in various cell types, including T cells, fibroblasts, and activated endothelial cells, and is notably present in tumor tissues, where it can contribute to tumor progression and immune evasion (3).

### 1.3 Aims of this review

This review aims to provide a comprehensive analysis of the TIME in PDAC and the roles of Gal-9 within this context. PDAC is known for its poor prognosis and resistance to conventional therapies, largely due to its complex TIME, which includes various immune cells, cytokines, cancer-associated fibroblasts (CAFs), and the extracellular matrix. Understanding the interactions and signaling pathways within this microenvironment is crucial for identifying new therapeutic targets. This review will detail the structure and function of Gal-9, highlighting its dual role in immune modulation and tumor pathogenesis, and its specific contributions to PDAC and other cancers. Additionally, the review will explore the potential of Gal-9 as a therapeutic target in immunotherapy, addressing current challenges and the promise of combination strategies to enhance treatment efficacy and overcome resistance. By integrating insights into PDAC biology, Gal-9 functions, and immunotherapeutic approaches, this review seeks to offer a detailed understanding of potential advancements in PDAC treatment.

## 2 Expression of Gal-9 in PDAC is dependent on tumor stage

Gal-9 has been extensively studied in the context of pancreatic cancer, but its role at different stages of the disease remains unclear, largely due to the challenges associated with late diagnosis (7). Histopathological studies have shown that Gal-9 is highly expressed in aggressive tumors (8, 9). In muscle-invasive bladder cancer, infiltration with Gal-9-expressing tumor-associated macrophages (TAMs) correlated with increased Treg cell numbers and poorer survival outcomes. In PDAC Gal-9 expression is significantly elevated in the tumor compared to normal tissue. Human cancer cells, particularly from aggressive tumors like PDAC, secrete Gal-9 in response to interactions with T lymphocytes, which leads to suppression of the immune response through mechanisms such as inhibition of T cell function and macrophage programming.

The serum levels of Gal-9 have been found to be a potential biomarker for PDAC. Elevated levels of Gal-9 in the serum can distinguish PDAC from healthy tissue and are prognostic for advanced stages of the disease (10, 11). Recent research has shown that Gal-9 expression and Treg levels increase in both the blood and pancreas of KC mice in correlation with the progression of precancerous lesions. In addition, Gal-9 expression was highly expressed in the early metaplastic stage in pancreatic intraepithelial neoplasm (PanIN), highlighting a potential role for Gal-9 in the early stages of PDAC. During these early stages, Gal-9 may facilitate immune evasion by recruiting regulatory T cells (Tregs) and suppressing anti-tumor immune responses (8). Gal-9’s interaction with the extracellular matrix (ECM) also plays a significant role in shaping the TME, influencing cell adhesion, migration, and signaling pathways that support tumor growth and metastasis (12, 13). As pancreatic ductal adenocarcinoma (PDAC) progresses, Gal-9 plays a critical role in establishing an immunosuppressive tumor microenvironment by driving macrophage polarization toward a protumoral M2 phenotype and suppressing cytotoxic T-cell activity. Furthermore, Gal-9 expression on blood-derived CD4+ and CD8+ T cells has been found to correlate with tumor size, lymph node metastasis, and UICC stage. Particularly, patients with larger tumor sizes exhibit significantly higher Gal-9 expression levels on CD4+ T cells (11). This makes Gal-9 not only a crucial player in the TME of PDAC but also a valuable marker for diagnosis and prognosis.

Moreover, expression and function of Gal-9 are dynamically regulated in response to various physiological stimuli. For example, endothelial cells can alter their expression of Gal-9 upon activation, which is significant in the context of tumor vasculature and angiogenesis (14). Such regulation ensures that Gal-9 can adapt to the changing microenvironment, thereby sustaining its role in immune modulation and tumor progression. Understanding the precise molecular structure and function of Gal-9, alongside its cellular localization, is critical for developing targeted therapies that can modulate its activity in cancer and other diseases.

## 3 Role of Galectin-9 in the tumor immune microenvironment of PDAC

TIME in PDAC is a complex and dynamic system comprising various immune cells and cytokines that play crucial roles in tumor progression, immune evasion, and therapy resistance. Effector T cells are notoriously inactive in PDAC. Instead, key components include TAMs, MDSCs, and Tregs shaping an immunosuppressive TIME. TAMs display high cellular plasticity that can be broadly categorized into two subtypes: M1 and M2 macrophages. M1 macrophages are generally pro-inflammatory and possess anti-tumor activity, whereas M2 macrophages are associated with anti-inflammatory responses and tumor progression.

### 3.1 Interaction of Gal-9 with T cells

Gal-9 is a negative regulator of anti-tumor immunity, suppressing the immune response by targeting T cells and inducing Tregs, which are known for their immunosuppressive capabilities in the TME (15). The suppression of effector T cell activity by Gal-9 is crucial in the context of PDAC, where the TME is notoriously immunosuppressive.

One of the critical mechanisms by which Gal-9 facilitates immune evasion in tumors is through its interaction with the immune checkpoint receptor Tim-3 on T cells. The Gal-9/Tim-3 pathway inhibits T cell function and induces apoptosis of effector T cells, thereby reducing the anti-tumor immune response and contributing to the creation of an immunosuppressive tumor microenvironment (9). In addition to Tim-3, Gal-9 binds to PD-1 which promotes the survival of exhausted T cell populations. (16). Gal-9 can also specifically interact with V-domain Ig-containing suppressor of T cell activation (VISTA), adding another layer to Gal-9’s role in immune suppression (9, 17). In the context of acute myeloid leukemia (AML), VISTA binds to Gal-9 secreted by AML cells as a ligand. Importantly, soluble VISTA released by AML cells enhances the effect of Gal-9, likely by forming multiprotein complexes on the surface of T cells and possibly creating a molecular barrier. These events cause changes in the plasma membrane potential of T cells, leading to the activation of granzyme B inside cytotoxic T cells and resulting in apoptosis (17). The immune-evasive effects of Gal-9 are also associated with other immunoregulatory molecules such as the amino acid L-kynurenine (LKU), which also suppresses anti-cancer T cell functions (18). This cooperation is also highly relevant to PDAC since IDO1, the enzyme which regulates LKU generation, is expressed by pancreatic cancer cells (19).

Further research has demonstrated that Gal-9 and VISTA co-expression on T cells is associated with a terminally exhausted phenotype, particularly noted in HIV-1 patients and virus associated tumors (20, 21). This phenotype is characterized by impaired effector functions and the upregulation of exhaustion markers such as EOMES, Blimp-1, and Glut-1. Gal-9 and VISTA co-expression leads to a profound reduction in T cell activity, significantly attenuating the immune response against tumors. This exhausted T cell phenotype includes effector T cells (CD45RA+, CD45RO-/lo, CD62L-, CD27lo) with high T-bet expression, indicating a dysfunctional state that is likely mirrored in tumor immunology. (9, 21, 22).

Interaction of Gal-9 with CD44 enhances the stability and function of adaptive regulatory T cells, reinforcing immunosuppression within the tumor microenvironment through TGF- β and Smad3 activation (23).

These findings suggest that the interaction between Gal-9 and VISTA might also contribute to immune evasion in solid tumors, reinforcing the immunosuppressive environment and promoting tumor progression (9, 21).

### 3.2 Interactions of Gal-9 with myeloid cells

Gal-9 expression in PDAC is elevated not only in tumor cells but also in myeloid cells, including TAMs and MDSCs, particularly in areas adjacent to tumors (8, 11, 24). This upregulation correlates with poor prognosis, as Gal-9 contributes to tumor growth, immune evasion, and the modulation of myeloid cell behavior within the tumor microenvironment (25).

#### 3.2.1 TAMs

In PDAC, TAMs are predominantly skewed towards an M2-like phenotype, contributing to an immunosuppressive environment that supports tumor growth and metastasis. Key signaling molecules such as TGF-β and CSF-1 play pivotal roles in the reprogramming of TAMs towards the M2 subtype. The ability of Gal-9 to polarize macrophages into an M2-like phenotype may significantly contribute to the immunosuppressive environment in PDAC (11, 26). An integrated analysis of transcriptomic datasets, including bulk and single cell RNA-seq data, identified Gal-9 as the primary component in cross-talk between TAMs and other cells. (27) While M2 macrophages contribute to tissue repair in normal physiology, in cancer, they promote tumor growth by suppressing immune responses In PDAC, Gal-9 interacts with Dectin-1 on macrophages, potentially driving a tolerogenic state that supports tumor progression. Notably, blocking Dectin-1 signaling in a PDAC mouse model reduced the infiltration of immunosuppressive CD206+ macrophages (24).

#### 3.2.2 MDSCs

MDSCs are a heterogeneous group of immature myeloid cells with potent immunosuppressive functions (28). In PDAC, MDSCs directly induce Treg cells through cell-cell interactions, while Treg cells reciprocally influence MDSC survival and proliferation (29). These interactions have been observed at multiple stages of cancer progression (29). Interestingly, while Gal-9 polarizes macrophages towards an M2 phenotype, high levels of LGALS9 (Gal-9) mRNA correlated with reduced expression of the M2 markers CD163 and CD206, but with elevated expression of the MDSC marker CD15 (11). This suggests that Gal-9 skews macrophages into a unique, partially polarized state that balances immunosuppression and support for tumor growth.

### 3.3 Targeting Galectin-9 in PDAC

PDAC presents numerous challenges in its treatment, largely due to inherent barriers and resistance mechanisms that reduce the efficacy of both chemotherapy and immunotherapy (30, 31). Particularly, the dense stromal environment of PDAC acts as a physical barrier. This fibrotic environment can inhibit angiogenesis, resulting in a sparse and dysfunctional vascular network (32). Consequently, the delivery of chemotherapeutic agents to the tumor cells is limited. Additionally, the tumor immune microenvironment (TIME) supports various immunosuppressive mechanisms that protect the cancer cells from immune attack, leading to resistance against immunotherapies (33, 34).

One of the primary approaches in immunotherapy is the use of immune checkpoint inhibitors (ICI), such as PD-(L)1 and CTLA-4 inhibitors, that target effector T cells. These inhibitors have revolutionized the treatment of various cancers by blocking pathways that inhibit T cell activation, thus enhancing the immune system’s ability to attack tumor cells. In cancers such as melanoma and lung cancer, these treatments have shown substantial success. However, their effectiveness in PDAC has been limited as the immunosuppressive TIME hampers the efficacy of these checkpoint inhibitors (16, 35, 36). A detailed comparison of Gal-9 with other immune checkpoints in PDAC, including mechanisms, roles, and clinical implications, is summarized in **Table 1**. Consequently, these traditional ICIs have shown limited success in clinical trials for PDAC patients, underscoring the need for alternative therapeutic targets (48).

**Table 1:** Comparison of Galectin-9 with other immune checkpoints in PDAC.

Immune Checkpoint	Mechanism	Role in PDAC	Clinical Implications	Similarities & Differences to Gal-9	Refs
Galectin-9 (Gal-9)	Binds to TIM-3 and VISTA on T cells, inducing apoptosis and promoting T cell exhaustion. Polarizes macrophages toward M2 macrophages, enhancing immunosuppression.	High expression correlates with poor prognosis. Promotes an immunosuppressive tumor microenvironment. Gal-9 levels can differentiate PDAC from benign conditions.	Targeting Gal-9 with anti-PD-L1 therapy shows enhanced tumor growth inhibition in preclinical models.	Unique Features: Directly interacts with TIM-3 and/or VISTA to induce T cell exhaustion and plays a role in macrophages polarisation	(11, 17, 24, 37)
PD-1/PD-L1	PD-L1 on tumor cells/APCs binds to PD-1 on T cells, inhibiting activation and proliferation.	Overexpression in PDAC contributes to T cell exhaustion and immune evasion. PD-L1 presence correlates with poor patient outcomes.	Anti-PD-1/PD-L1 therapies have limited efficacy in PDAC due to the highly suppressive tumor microenvironment (TME).	Similarities: Both Gal-9 and PD-L1 suppress T cell function and are associated with poor prognosis. Differences: PD-L1 expression is mediated by the MLL1-H3K4me3 Axis.	(38–40)
CTLA-4	Competes with CD28 for B7 ligands on APCs, inhibiting T cell activation.	Associated with Treg activity in PDAC, contributing to immune suppression.	Anti-CTLA-4 therapies have shown limited success in PDAC due to the strong immunosuppressive signals.	Similarities: Both checkpoints suppress T cell activation. Differences: CTLA-4’s main role is in inhibiting early T cell activation, whereas Gal-9 affects already activated T cells.	(41, 42)
LAG-3	Inhibitory receptor binding to MHC class II molecules, reducing T cell activation.	Expressed on exhausted T cells in the PDAC microenvironment, contributing to immune evasion.	Targeting LAG-3 may enhance the effectiveness of other checkpoint inhibitors in combination therapies.	Similarities: Like Gal-9, LAG-3 is expressed on exhausted T cells and contributes to immune evasion. Differences: LAG-3 does not modulate macrophages or use ligand-specific signaling.	(43, 44)
TIM-3	Inhibits T cell responses by binding to ligands (e.g., Gal-9), causing T cell apoptosis or functional impairment.	highly expressed in pancreatic cancer tissues and strongly associated with invasion, metastasis, and recurrence.	Combination therapies targeting TIM-3 and other checkpoints like PD-1 may reinvigorate anti-tumor immunity.	Similarities: Directly interacts with Gal-9 to suppress T cell function. Differences: TIM-3 relies on Gal-9 for its effects, while Gal-9 has additional macrophage-modulating properties.	(16, 45)
IDO (Indoleamine 2,3-Dioxygenase)	Depletes tryptophan and produces kynurenine, leading to local immune suppression and Treg differentiation.	IDO expression correlates with poor prognosis in PDAC by promoting an immunosuppressive environment.	IDO inhibitors show variable results in clinical trials but remain a focus for potential therapies.	Similarities: Both Gal-9 and IDO contribute to T cell exhaustion and immune suppression. Differences: IDO’s mechanism involves metabolic pathways, unlike Gal-9’s receptor-ligand binding.	(46, 47)

### 3.4 Immunotherapeutic potential of Galectin-9

Gal-9 has already shown significant therapeutic potential in various cancers. Enhanced expression of Gal-9 has been associated with improved overall survival in various solid cancers, including colon and hepatocellular cancer, as well as improved disease-free survival in gastric and non-small-cell lung cancer (49). Gal-9 mediates its tumor-suppressive effects through multiple mechanisms, including the induction of apoptosis in tumor cells, modulation of immune responses, and inhibition of metastasis. Particularly, increased levels of caspase-cleaved keratin 18 levels, as a measure of apoptosis, have been observed in Gal-9 treated colon cancer cells. Gal-9 dependent increase of caspase activity has also been observed in HCC cells *in vitro* and *in vivo* (50). Anti-metastatic potential of Gal-9 has been observed in triple negative breast cancer (TNBC), HCC (51) and melanoma (52). Additionally, in triple-negative breast (TNBC) cancer Gal-9 expression correlates with increased immune cell infiltration and positive PD-L1 expression on tumor cells. This dual role of promoting tumor cell death while modulating the immune environment makes Gal-9 a compelling target for therapeutic intervention. Furthermore, combining anti-Gal-9 therapy with anthracycline-based chemotherapy, such as doxorubicin, significantly enhances antitumor activity in breast cancer. This combination therapy leverages the complementary mechanisms of action of both treatments (53).

In the context of PDAC, preclinical studies have demonstrated that targeting Gal-9 can disrupt its interactions with Tim-3 and other immune modulators, thereby enhancing anti-tumor immune responses. This approach aims to mitigate the immunosuppressive effects of Gal-9, allowing the immune system to effectively target and eliminate cancer cells (8, 26). Moreover, the blockade of Gal-9 can potentially enhance the efficacy of other immunotherapeutic agents, such as PD-(L)1 and CTLA-4 inhibitors, by relieving the suppression of immune cell activity within the tumor microenvironment. Blockade of Gal-9 can be achieved with neutralizing antibodies or lactose (54–56).

Given the limitations of current immunotherapy in PDAC, targeting components of the TIME with nanomaterials can significantly enhance immunotherapeutic efficacy and optimize drug delivery outcomes. Therefore, combining anti-Gal-9 therapies with nanoparticle-based delivery systems may further enhance this approach in PDAC. Nanoparticles can precisely target tumor cells and modulate the immune response, providing a dual attack on the cancer. This method improves drug delivery, reduces off-target effects, and increases the overall efficacy of the treatment (34, 57). Early studies suggest that these combination therapies may improve patient outcomes by reducing tumor growth and metastasis, representing a novel and promising approach to overcoming the inherent barriers in PDAC treatment and enhancing the overall effectiveness of existing therapies (8, 16). Additionally, in a mouse model, T7 peptide-decorated exosome-based nanoparticles delivering Gal-9 siRNA significantly inhibit glioblastoma and enhance antitumor immunity.

Overall, the therapeutic potential of Gal-9 in cancer treatment is substantial, particularly in PDAC where traditional therapies have limited effectiveness. The development of anti-Gal-9 antibodies and other Gal-9-targeted therapies holds promise for improving outcomes in patients with PDAC and other malignancies by harnessing the immune system’s power to combat cancer. Continued research is necessary to fully understand the mechanisms by which Gal-9 modulates tumor and immune cell interactions and to translate these findings into effective clinical treatments. Despite the significant therapeutic potential of Gal-9, a search in ClinicalTrials.gov revealed only two current clinical trials that are interventional (see **Table 2**), while the other registered trials involving Gal-9 are observational (see **Table 3**). This underscores the need for expanded research and a greater number of clinical trials to fully explore and harness Gal-9’s therapeutic capabilities. Increasing the focus on Gal-9 in clinical settings is crucial for unlocking its potential in cancer treatment and other diseases.

**Table 2:** Summary of interventional studies on Gal-9 in cancer research.

Name	Interventions	Cancer types	Phase	Status	Clinical trials	Completion date
LYT-200: monoclonal antibody (mAb), targeting galectin-9 protein	LYT-200 as a Single Agent and in Combination with Chemotherapy	Pancreatic cancer, metastatic cancer	1 & 2	Recruiting	NCT04666688	Jan. 2025
LYT-200: monoclonal antibody (mAb), targeting galectin-9 protein	LYT-200 as a Single Agent and in Combination with Chemotherapy	Relapsed/Refractory Acute Myeloid Leukaemia	1	Recruiting	NCT05829226	Mai. 2025

**Table 3:** Summary of observational studies on Gal-9 in cancer research.

Observations	Cancer types	Status	Clinical trials	Completion date
Characterisation of TIM-3/Gal-9 Immune Checkpoints	B Cell Lymphomas	Completed	NCT05133505	Nov. 2021
Determination of Immune Check Point Levels including PD-1, TIM-3, LAG-3 PD-L1 and Gal-9	Gastrointestinal Cancer	Completed	NCT04566848	Jan. 2022
Evaluation of the Role of Immune Checkpoints including PD-1, TIM-3, LAG-3 PD-L1 and Gal-9 in Response to Neoadjuvant Therapy	Breast Cancer	Completed	NCT05519397	Oct. 2022
Measurement of Immune Checkpoints including PD-1, TIM-3, LAG-3 PD-L1 and Gal-9 in Intraabdominal Ascites Fluid	Advanced Colorectal Cancer	Completed	NCT04540159	Dec. 2022
Evaluation of the Relationship Between Stage I-II Breast Cancer Subtypes and Soluble Immune Checkpoints including PD-1, TIM-3, LAG-3 PD-L1 and Gal-9	Breast Cancer Subtypes	Completed	NCT05460702	Dec. 2022

### 3.5 Immunotherapeutic combination strategies

Combining various immunotherapeutic agents can help overcome the limitations of single-agent therapies and address mechanisms of resistance that tumors often develop. Interestingly, a phase II study in PDAC using combination of radiation therapy and anti-PD-1 or anti-CTLA-4 antibodies, indicated increased protein levels of circulating FasL, Gal-1 and Gal-9. Conversely, Gal-9 and PD-L1 combination treatment inhibits PDAC tumor progression in a murine PDAC model (37).

A promising strategy might thus involve the simultaneous blockade of Gal-9, Tim-3 or other ICIs including other galectins, to prevent compensatory mechanisms and resistance. Tumors often exploit multiple pathways to evade immune detection and destruction, so targeting several of these pathways at once can be more effective. For example, blocking Gal-9 and Tim-3 can disrupt the immunosuppressive signals that inhibit T-cell function, while ICIs like anti-PD-1 can further enhance T-cell activity against the tumor. However, ICI combination therapy can increase the risk of immune-related adverse events. In a meta-analysis of 18 studies spanning 10 cancer types, combination ICI therapy was linked to a modestly increased risk of all-grade adverse events and a significantly higher risk of grade 3 or more severe adverse events compared to ICI monotherapy (58).

Another innovative approach is the use of anti-Gal-9 in combination with reprogrammed macrophages or chimeric antigen receptor macrophages (CAR-M). These therapies aim to transform TAMs, which typically promote tumor growth and suppress immune responses, into a phenotype that supports anti-tumor immunity. Reprogrammed macrophages or CAR-M can enhance the phagocytosis of cancer cells and stimulate other immune cells to attack the tumor (59–62). When combined with anti-Gal-9, these therapies can further disrupt the immunosuppressive environment created by the tumor and enhance the overall immune response.

These combination strategies highlight the potential of leveraging multiple facets of the immune system to combat cancer more effectively. By targeting different pathways and using innovative delivery methods, these approaches aim to improve patient outcomes in PDAC and other challenging cancers. Continued research and clinical trials are necessary to optimize these combinations and determine the most effective protocols for different patient populations.

## 4 Conclusion

**Figure 1:**
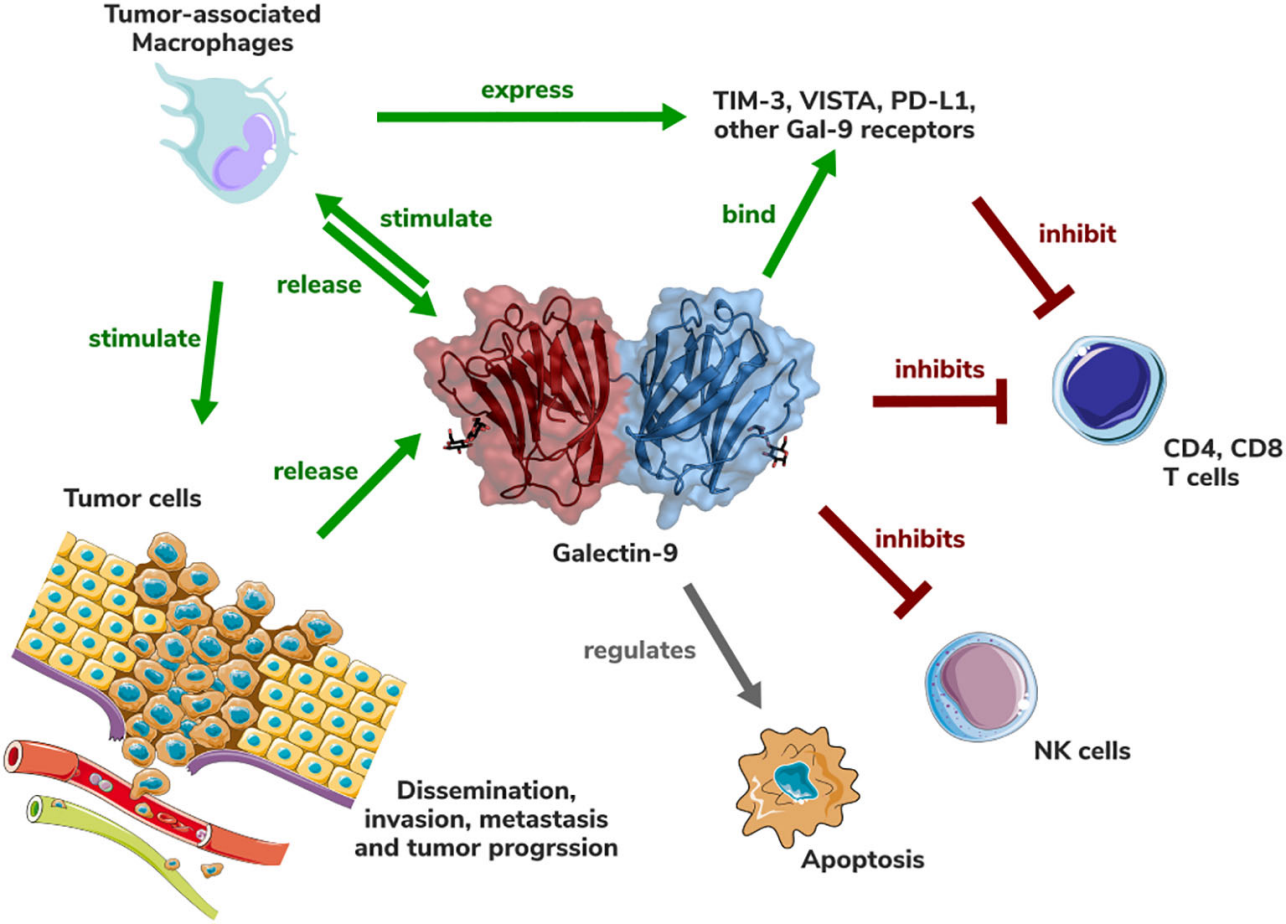
Schematic representation of the role of Galectin-9 in the PDAC tumor (immune) microenvironment. Gal-9 structure (PDB accession number: 3WV6) is shown in cartoon and surface representation.

Gal-9 emerges as a pivotal modulator within the tumor immune microenvironment (TME) of pancreatic ductal adenocarcinoma (PDAC), orchestrating immunosuppressive mechanisms that promote tumor progression and therapy resistance **Figure 1**. Its multifaceted roles in regulating immune responses, including the induction of regulatory T cells (Tregs), polarization of TAMs to an M2-like phenotype, and interaction with immune checkpoints such as Tim-3, underscore its centrality in PDAC’s notoriously challenging TME. Elevated Gal-9 expression in PDAC, coupled with its association with poor prognosis, reinforces its potential as a prognostic biomarker and therapeutic target.

A critical question in targeting Gal-9 is whether its opposing roles—promoting immune suppression in the TME while also exhibiting immune-stimulatory and anti-metastatic effects under certain contexts—can be therapeutically separated. Current evidence suggests that these divergent outcomes are highly dependent on the tumor type, stage, and specific microenvironmental factors. In PDAC, Gal-9 predominantly acts as an immune suppressor, and therapeutic strategies must focus on blocking its immunosuppressive interactions, particularly with Tim-3, Tregs, and TAMs. However, a more nuanced understanding of how Gal-9’s dual roles are regulated will be essential for developing precise interventions that maximize therapeutic benefits while minimizing unintended consequences.

Therapeutic targeting of Gal-9 presents several opportunities. Blocking Gal-9 can enhance the efficacy of immune checkpoint inhibitors such as PD-(L)1 and CTLA-4 inhibitors, thereby overcoming resistance to immunotherapy in PDAC. Combining Gal-9 inhibitors with advanced drug delivery systems, such as nanotechnology, holds potential for enhancing both the specificity and potency of treatments. Additionally, novel strategies like reprogramming TAMs or leveraging chimeric antigen receptor macrophages (CAR-M) in conjunction with Gal-9 inhibition could further disrupt the immunosuppressive TME and stimulate anti-tumor immunity. Early preclinical evidence is promising, but significant translational work is needed to validate these approaches in clinical settings.

In summary, Gal-9 represents a compelling and multifaceted therapeutic target in PDAC. To fully exploit its potential, future research must prioritize understanding the mechanisms underlying its dual roles and focus on refining therapeutic strategies to selectively neutralize its immunosuppressive functions. By targeting Gal-9, there is a tangible opportunity to overcome the inherent barriers of PDAC’s TME, paving the way for more effective and durable treatment options in this challenging malignancy.
